# The Facial Line and Angles in Prosthetic Dentistry

**Published:** 1897-11

**Authors:** W. E. Walker

**Affiliations:** Pass Christian, Miss.


### The Facial Line and Angles in Prosthetic Dentistry.

BY DR. W. E. WALKER, D.D.S., PASS CHRISTIAN, MISS.

Abstract of paper read before the Southern Dental Association, Old Point Comfort, August, 1897.

This paper is designated as No. 3, being a continuation of the line of thought presented in paper No. 1, published in *Cosmos*, January, 1896, and No. 2, published in the *Journal* of the American Medical Association, February 6, 1897. The importance of the study of the teeth, in connection with the reproduction of the maxillary movements in mastication, is pointed out by Dr. Broomill (see *Cosmos*, January, 1897), who says: "Each individual tooth in itself tells of the nature of the movements in the inferior maxilla during mastication, and these inherent movements, established in accordance with the formation of the natural teeth, should not be interfered with in prosthesis."

In the short movements of mastication the condyle on the side toward which the jaw is advancing travels forward and downward on a line which being extended till it intersects the facial line, forms at the point of intersection an angle which varies with and bears a definite relation to the morphology of the morsel surfaces of the teeth. As this angle varies very considerably, not only in different individuals, but quite frequently in the two condyles of the individual, it is important, *first*, to be able to determine accurately the direction of the movement of the condyle—the condyle path as I have called it—compared with the facial line, and the plane of occlusion, and *second*, to be able to exactly reproduce the movements of the jaw in an articulator carrying the substitutes for lost dentures. The path traversed by the condyle, extended till it intersects the facial line, forms what I have termed the condyle facial angle. The difference between this angle and that formed by the alveolar plane and the facial line, is the condyle alveolar angle. The importance of the study of these lines and angles is pointed out by Dr. Broomill in the article before referred to, with special reference to ascertaining the tooth-line level, which, Dr. Broomill says, is always horizontal to the basal line of the facial angle.

To measure the condyle movement and these various lines and angles, I have devised an instrument which I have called the facial clinometer, which I will demonstrate in the clinics. The study of the facial line and angles is not only a matter of scientific interest, but is of practical value to the dental and oral prosthetist who desires to receive that scientific accuracy essential to perfection in the practical value of and comfort to the wearer of dental prosthetic appliances.

In the discussion of this paper DR. GRANT MOLYNEAUX said that he had read Dr. Walker's series of papers and appreciated what he was doing in the cause of prosthetic dentistry, but could not agree with him on many points. He had adopted Dr. Bonwill's system of articulation because it was a perfect system, enabling us to teach the student by fixed and certain rules. He himself had carried out this system a thousand times or more and could not differ from him. He did not believe in destruction without a corresponding construction, and until Dr. Walker could give us something better than Dr. Bonwill's he would continue to follow the latter.

Dr. Beadles said that he would be glad if Dr. Walker would state his side of the question clearly in a few words, and let Dr. Molyneaux do the same, that it might be seen wherein the difference between the two systems lay.

In response Dr. Walker said that no one was more ready than he to give credit to Dr. Bonwill for his pioneer work in the line of articulating artifical teeth. Dr. Bonwill had devised an apparatus for the purpose of attaining certain conditions—a balancing articulation especially. In his own practical work in the laboratory he (Dr. Walker) had found that Dr. Bonwill's apparatus—his anatomical articulator—was faulty in that portion of the mechanism which controls the condyle movement, in that it reproduces a condyle movement which is forward, that is, parallel with the plane of occlusion. That this is not correct is seen by placing casts of the natural teeth in the Bonwill articulator, when it will be found that the cusps of the molars will interfere with placing the incisors in the biting position, and also with placing the lower jaw in the lateral position as for grinding. If casts of natural teeth cannot be placed in position to bite and grind in an articulator, there is something wrong in the machine. He found that the fault lay in the necessity for an adjustable angle, by means of which the actual movements of the jaw can be reproduced. Being asked if he would demonstrate what he claimed, Dr. Walker replied that he could, and would do so in the clinics of the present meeting.

Dr. W. T. Arrington said that he had for many years confined himself to the old methods of articulation. He had tried to understand Dr. Bonwill's methods, but in vain. He had also failed to comprehend Dr. Walker's papers, but from his last remark he was forced to believe that Dr. Walker's ideas, as applied in his articulator, were as far above Dr. Bonwill's as Dr. Bonwill's were above the old friend plaster articulator.

Dr. J. T. Crawford said: We are still fighting the battle I engaged in eighteen years ago, when I urged the importance of a knowledge of anatomy applied to the articulation of artifical teeth. I regard Dr. Bonwill's work as very valuable, but if there is anything in the respective claims made, Dr. Bonwill's articulator will not stand the test. Dr. Walker has made a point on Dr. Bonwill in the reproduction of the movement of the lower jaw, as it sweeps downward as well as forward. If there is anything in Dr. Bonwill's claim for the necessity of an anatomical articulator, there is far greater necessity for Dr. Walker's physiological articulator, for it is essential that we should be able to reproduce the true movements of the lower jaw, in order to secure the points of contact simultaneously at the three angles of a triangle. If Dr. Bonwill and Dr. Walker, conjointly, have accomplished that, they will have lifted prosthetic dentistry from the slum into which it had fallen; rescued it from the position of an incubus upon the profession.

Dr. J. D. Patterson spoke of the acquired movements of the jaw common to persons of advanced age who had lost the natural organs, and the impropriety of attempting to normal overbite and articulation of youth and middle age to such cases.

Dr. Crawford said it was the duty of the dentist in every case to give a correct occlusion and a serviceable articulation.

Dr. Molyneaux: Dr. Bonwill never intended to convey the idea that the relation between the overbite and the curvature of line of occlusion was the same in every case. Conditions are modified by advancing age. I deny that in Dr. Bonwill's articulator the lower jaw does not move downward and outward exactly as does the human jaw. All imitations of or attempts to improve upon the Bonwill articulator have been failures. I had one of the first Bonwill articulators that appeared, and having mastered his system, I unhesitatingly pronounce it the greatest boon ever bestowed upon prosthetic dentistry. I have no desire to antagonize Dr. Walker. I would be glad to have him show us a better way, but I do not think he has proved his position yet.

Dr. Walker, in closing the discussion, said that the essential thing was to be enabled to reproduce the movements of the jaw habitual to the patient in hand, and to be enabled to so arrange artificial teeth that they will articulate in accordance with those movements. The object of the physiological articulator is to reproduce the habitual jaw movements of the patient. With my articulator I know when the articulation is correct. If it is not right in the mouth, my articulator shows me how to correct it.

